# Neighborhood-targeted and case-triggered use of a single dose of oral cholera vaccine in an urban setting: Feasibility and vaccine coverage

**DOI:** 10.1371/journal.pntd.0005652

**Published:** 2017-06-08

**Authors:** Lucy A. Parker, John Rumunu, Christine Jamet, Yona Kenyi, Richard Laku Lino, Joseph F. Wamala, Allan M. Mpairwe, Vincent Muller, Augusto E. Llosa, Florent Uzzeni, Francisco J. Luquero, Iza Ciglenecki, Andrew S. Azman

**Affiliations:** 1 Médecins Sans Frontières, Geneva, Switzerland; 2 CIBER Epidemiología y Salud Pública, Department of Public Health, Universidad Miguel Hernández, Alicante, Spain; 3 Republic of South Sudan Ministry of Health, Juba, South Sudan; 4 World Health Organization, Juba, South Sudan; 5 Epicentre, Paris, France; 6 Department of International Health, Johns Hopkins Bloomberg School of Public Health, Baltimore, Maryland, United States of America; 7 Department of Epidemiology, Johns Hopkins Bloomberg School of Public Health, Baltimore, Maryland, United States of America; The Johns Hopkins University, UNITED STATES

## Abstract

**Introduction:**

In June 2015, a cholera outbreak was declared in Juba, South Sudan. In addition to standard outbreak control measures, oral cholera vaccine (OCV) was proposed. As sufficient doses to cover the at-risk population were unavailable, a campaign using half the standard dosing regimen (one-dose) targeted high-risk neighborhoods and groups including neighbors of suspected cases. Here we report the operational details of this first public health use of a single-dose regimen of OCV and illustrate the feasibility of conducting highly targeted vaccination campaigns in an urban area.

**Methodology/Principal findings:**

Neighborhoods of the city were prioritized for vaccination based on cumulative attack rates, active transmission and local knowledge of known cholera risk factors. OCV was offered to all persons older than 12 months at 20 fixed sites and to select groups, including neighbors of cholera cases after the main campaign (‘case-triggered’ interventions), through mobile teams. Vaccination coverage was estimated by multi-stage surveys using spatial sampling techniques. 162,377 individuals received a single-dose of OCV in the targeted neighborhoods. In these neighborhoods vaccine coverage was 68.8% (95% Confidence Interval (CI), 64.0–73.7) and was highest among children ages 5–14 years (90.0%, 95% CI 85.7–94.3), with adult men being less likely to be vaccinated than adult women (Relative Risk 0.81, 95% CI: 0.68–0.96). In the case-triggered interventions, each lasting 1–2 days, coverage varied (range: 30–87%) with an average of 51.0% (95% CI 41.7–60.3).

**Conclusions/Significance:**

Vaccine supply constraints and the complex realities where cholera outbreaks occur may warrant the use of flexible alternative vaccination strategies, including highly-targeted vaccination campaigns and single-dose regimens. We showed that such campaigns are feasible. Additional work is needed to understand how and when to use different strategies to best protect populations against epidemic cholera.

## Author summary

Oral cholera vaccine (OCV) is becoming part of the standard cholera-control toolkit, although experience in deploying OCV is limited. Adapting vaccination strategies to the global availability of vaccines and the local context (i.e., population movement, security constraints, etc.) is key to maximize the impact of OCV as a cholera-control tool. Here we describe the operational details of the first field use of a single-dose of OCV, which was deployed in a targeted manner, both at high-risk neighborhoods and then to neighbors of suspected cases after the main OCV campaign when sporadic cholera case reports continued. We show that it is feasible to conduct micro- and macro-targeted vaccination campaigns in urban areas like Juba with moderate to high coverage and without social unrest due to vaccinating some groups and not others. Flexible and context-adapted OCV dosing regimens and strategies should be considered in future deployments of the vaccine.

## Introduction

Oral cholera vaccine (OCV) is an effective tool to prevent and control cholera both in endemic settings and in response to outbreaks [1,2]. On 23-June-2015, the Republic of South Sudan Ministry of Health (MoH) declared a cholera outbreak in Juba, the nation’s capital. Initial cases were traced back to 18-May in the United Nations Protection of Civilians Camp, where approximately 28,000 internally displaced people (IDP) resided. By the time the epidemic was declared, cases had been confirmed throughout the city and public health officials believed that Juba was at risk for a large cholera outbreak, with the threat of spread to other areas of the country. The MoH convened the National Cholera Taskforce to guide a comprehensive outbreak response involving case management, water and sanitation interventions, health education and hygiene promotion. Following a situation assessment and in light of the 2014 cholera outbreak with 6,269 reported cases and 156 deaths in multiple areas of the country [3], the MoH, supported by Médecins sans Frontières (MSF), decided to integrate OCV into the cholera response in Juba.

Only 270,000 doses were released from the global emergency OCV stockpile due to severe supply limitations despite the much larger at-risk population (500,000–1,000,000 people). The MoH decided to use an off-label, single-dose, regimen in a targeted vaccination campaign. The rationale was based on preliminary results from a large randomized clinical trial [4] demonstrating significant 1-dose protection in Bangladesh, immunogenicity studies [5] and modelling analyses [6] showing that even with a significantly less effective one-dose regimen, one-dose campaigns may save more lives than their two-dose counterparts when supply is limited. The goal was to quickly provide protection to the maximum number of people at highest risk with a, perhaps less effective, single-dose regimen, rather than covering half the number of people with the standard two-doses. The possibility of providing a second dose, to potentially increase the effectiveness and extend the duration protection, when supplies were available was not ruled out.

Two targeted vaccination approaches were used with an aim to halt transmission and shorten the duration of the epidemic. First, OCV was targeted to neighborhoods with evidence of significant transmission just prior to the campaign and vulnerable groups at higher risk of cholera including IDPs, prisoners and health care workers. After the main campaign, sporadic case reports continued, mostly in unvaccinated neighborhoods. Given that the risk of cholera has been shown to be highly elevated among those living around a cholera case in the days after the case presents for care at a clinic [7,8], the remaining vaccine was delivered to neighbors living around suspected cholera cases together with water sanitation and hygiene measures (case-triggered interventions). Further details related to the decision-making process, timeline and vaccine effectiveness are described in detail elsewhere [9,10]

Here, we describe the operational details and vaccine coverage of these spatially targeted OCV delivery approaches, from campaigns that represent, to our knowledge, the first field-use of a single-dose of OCV in response to an epidemic, and the first use of case-triggered cholera interventions including OCV. We explore vaccine uptake in the different areas targeted, including neighborhoods and smaller areas around the households of suspected cases, identify difficult-to-reach population groups and discuss alternative campaign strategies to improve vaccine coverage.

## Methods

### Target population

The campaign setting was particularly challenging amid a humanitarian crisis with significant population displacement. Accurate population estimates were not available, so we used two approaches to define the target populations when planning the campaigns. First, we extrapolated population estimates for different areas of the city using data from the latest population census in 2009 [11]. This census was conducted prior to independence and civil war and extrapolated population size estimates are believed to vastly underestimate the true population size. Next, we used estimates of the number of built structures in each area from recent digitized satellite images (http://wiki.openstreetmap.org/wiki/WikiProject_South_Sudan). We initially assumed 70% of the structures with a roof footprint from 5 to 250 m^2^ were residential and that each had an average of 6 people [11].

**Fig 1:**
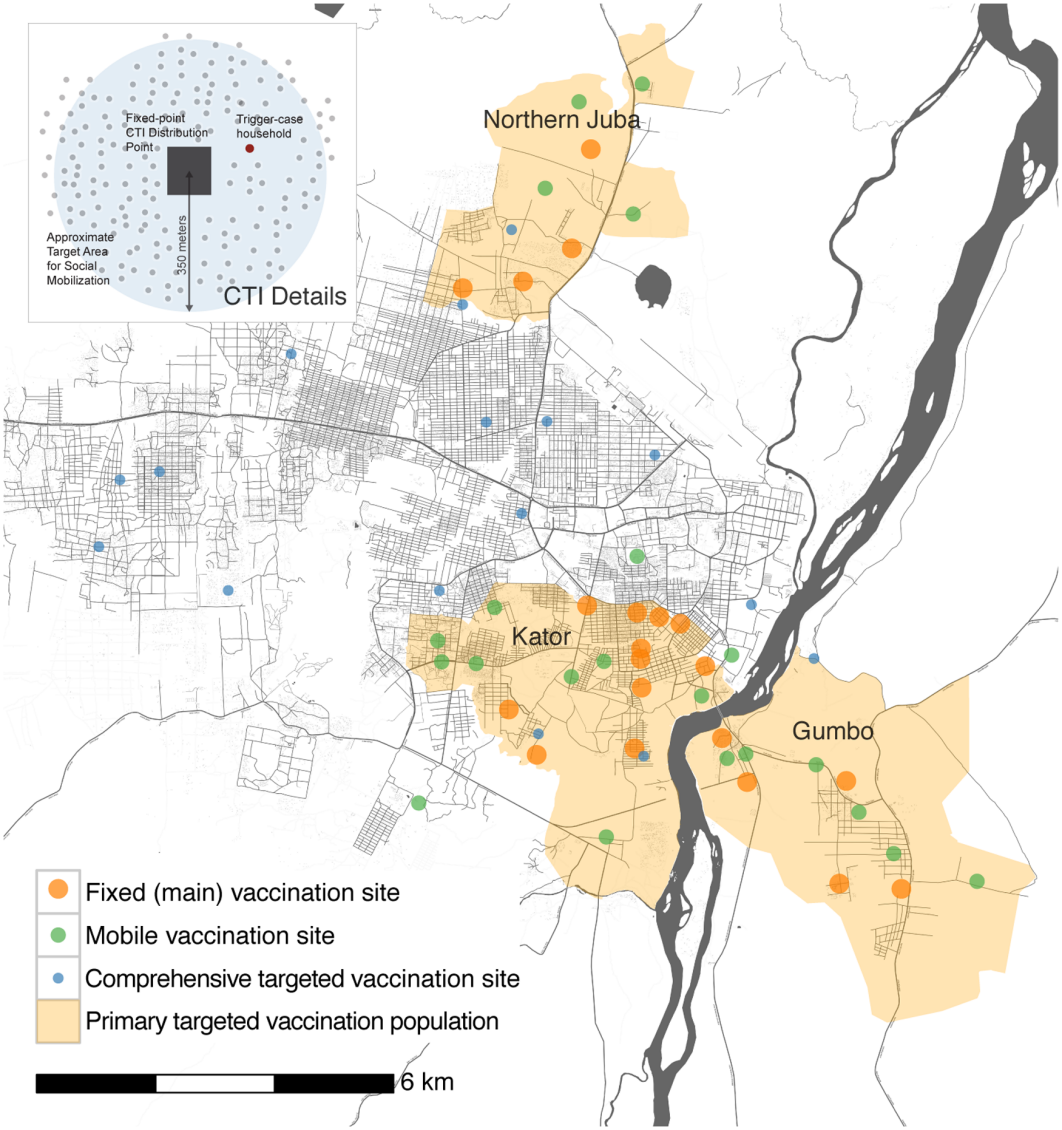
Overview of vaccination areas in Juba. Sub-panel on top left illustrates the case-triggered comprehensive targeted intervention (CTI) approach.

In this rapidly evolving epidemic, the decisions of where to target vaccine were based on the most up to date cumulative attack rates, recent incidence and local knowledge of known cholera risk factors. Three main areas were identified, using unofficial boundaries and referred to here as Kator, Northern Juba and Gumbo, with combined population estimates ranging from 53,543, from census data, to 368,136 from satellite imagery (Fig 1). The three areas differed greatly by socio-demographics. Kator is a densely populated area including a semi-commercial part of the city that had experienced a spike of suspected cholera cases just prior to the campaign and a large slum-like area bordering the Nile river. Northern Juba is a fairly-isolated settlement next to a large military base, predominantly inhabited by military members and their families. Gumbo is an area with moderate population density on the south-eastern side of the river Nile with predominantly poor-housing and persistent notification of cholera cases preceding the campaign. In addition to the three targeted areas of the city, IDPs living in informal camps, inmates in Juba’s prison, health care workers and residents living close to suspected cholera patients presenting after the main campaign were targeted by mobile teams.

### Neighborhood-targeted vaccination campaign

A single dose of OCV (Shanchol^®^, Shantha Biotechnics Ltd, Hyderabad, India) was offered to all persons older than 12 months presenting at vaccination sites, regardless of her/his area of residence. Twenty fixed vaccination sites operated from 08:00–17:00 from 31-July to 5-August each with a team of approximately 20 people per site (3–4 vaccinators, 3–4 individuals preparing the vaccine, 8–10 registrars filling out vaccine cards and tally sheets, 1 security guard, 2 health promoters and 1 team supervisor). As the number of individuals coming to the sites slowed (3-August), vaccination teams split into semi-mobile units and set up mini-vaccination sites to reach those not yet covered. Vaccines were stored under cold chain (2–8°C) using a refrigerated truck, transported to the vaccine site in their original Styrofoam box with icepacks and then used at ambient temperature the day of vaccination. We distributed a vaccination card to each vaccinee indicating her/his name, age, vaccination location, date of vaccination and vaccine lot number.

We avoided the widespread use of radio and other media to publicize the campaign due to concerns that offering limited vaccine only in selected parts of the city could spark civil unrest. Trained health promoters disseminated information regarding the campaign in the targeted communities, and members of the target populations were recruited to spread key messages using megaphones.

To assess vaccine coverage in the neighborhood-targeted (main) campaign, a random sample of the population living in each of the target areas was selected using a stratified spatial sampling approach, with households serving as the primary sampling unit. A total of 128 households, including all household members, were required in each of the three target areas to estimate each area-specific coverage with a precision of ±5%. Detailed methods for the coverage survey, conducted 9–14 August, are provided in S1 Text.

### Case-triggered comprehensive targeted interventions

From 13–26 August, after the main campaign but before cholera case reports had stopped within the city, OCV was included as part of a case-triggered comprehensive targeted intervention (CTI) approach. Vaccine was offered together with soap, water purification tablets, a leaflet on cholera prevention and health promotion by mobile teams made up of staff from multiple organizations, including the MoH, South Sudanese Red Cross, Oxfam and MSF. This activity required close coordination with multiple governmental and non-governmental actors to (1) detect and test suspected cases, (2) rapidly communicate results from cholera diagnostic tests, (3) locate the case’s household and decide on the location for intervention in conjunction with local leaders and (4) deploy the intervention in a timely manner.

Patients from Juba reporting to any of the cholera treatment centers or oral rehydration posts with a stool sample positive for cholera using the Crystal VC rapid diagnostic test (RDT), either directly or after a 4–6 hour enrichment in alkaline peptone water [12], were put on a list for CTI eligibility. Due to limited human resources, cases coming from areas that had not been covered in the neighborhood-targeted OCV campaign and those testing positive to the, more specific [12], enriched RDT were prioritized. When possible, teams also conducted CTI in previously vaccinated areas where it appeared that cholera transmission may have continued. These CTIs were also targeted to the homes of individuals who died of acute watery diarrhea, either in the community or in a health-facility, even if no stool sample had been tested.

The Juba County Health Department’s rapid response team and MSF staff travelled to the home of CTI-eligible suspected cases. Together with a community leader, they identified a suitable intervention site as close as possible to the home of the patient and recruited four community members: two to assist with security/crowd control and two for going door-to-door informing the neighbors about the intervention and encouraging residents to come to the sites. Volunteers were not informed of the specific rationale behind the location of intervention site (i.e. details of the suspect cholera case triggering the CTI) to respect the patients’ privacy, but they were given a specific geographic focal area. The site was typically set-up the following day by a 5-7-person team (1 site supervisor, 1–2 vaccinators, 1–2 registrars for completing vaccination cards and tally sheets and 2–3 people delivering the water/sanitation/hygiene intervention). All individuals coming to the site were eligible for OCV regardless of whether they had been vaccinated in the main vaccination campaign.

To assess vaccine coverage in each case-centered targeted intervention cluster, we selected 30 spatially random points within 350-meters (assumed as the catchment area of the interventions) of suspected case households (Fig 1, S1 Text). As with the population-based survey for the main campaign, the closest household to each GPS point was included in the survey, but instead of ascertaining the vaccination status of all individuals living in the household as done in the main coverage survey, one person was selected at random from those residing in (but not necessarily present at the time of the first survey visit) the household.

### Data collection and analysis

We collected data on age, sex, vaccination status (both verbal and confirmed with card) and reasons for non-vaccination (when applicable) for everyone included in the coverage surveys. We also collected household-level variables including the number of household members at the time of the campaign, the number of built structures included in each household and their spatial coordinates.

We estimated mean vaccination coverage and 95% confidence intervals for individual vaccination target areas (both neighborhoods and areas around case-triggered interventions) and for the entire target population. In secondary analyses, we estimated the coverage by age group, sex and distance to the closest vaccination site. Individuals with missing information on vaccination status were excluded from the analysis. Relative risks and 95% confidence intervals were estimated using a generalized linear model with a log link. All confidence interval estimates for vaccine coverage and relative risks took into account the survey design (clustering by household and vaccination area) using the *svy* commands in Stata 12.0 (College Station, TX, USA).

### Ethics

This was a public health intervention designed to prevent the spread of cholera, informed consent for participation was not required. The activities presented in this study were conducted as standard monitoring and evaluation exercises, thus approval from ethical review committees was not obtained. Although written informed consent was not solicited for the coverage surveys, all interviewees provided verbal consent and no identifiable information was collected other than household coordinates.

## Results

From 31-July to 26-August-2015, 162,377 people were vaccinated through targeted campaigns in Juba. 127,191 vaccines were distributed at fixed vaccination posts as part of a neighborhood-targeted approach from 31-July to 5-August-2015, 8,592 (6.1%) were distributed in informal IDP settlements. Mobile vaccination teams provided 1,011 doses (0.7%) in a local prison and 3,455 doses (2.5%) to healthcare workers. A further 22,128 people received the vaccine as part of 17 case-triggered CTI deployments from 13–26 August.

### Neighborhood-targeted vaccination campaign

Most of the vaccines (91,953 doses, 65.6%) were distributed in the targeted area of Kator. In the neighborhoods targeted in Northern Juba, 21,039 individuals received OCV, which was greater than the population estimated by both census and using satellite imagery (Table 1). Just over half of those receiving vaccine during the neighborhood-targeted campaign were male (71,945, 51.3%) and 75,638 (53.9%) were at least 15 years old (Table 2, S1 Table).

**Table 1:** Population size and vaccine coverage estimates for neighborhood-targeted vaccination campaign.

	Received OCV^1^	Population estimate from census		Population estimate from satellite images^2^		Population-based survey
	Doses	N	Administrative Vaccine Coverage^3^, (%)	N	Administrative Vaccine Coverage^3^, (%)	Vaccine Coverage^4^,% (95%CI)
**Kator**	91,953	48,470	190	126,540	73	70(63–77)
**Northern Juba**	21,039	906^5^	2322	19,425	108	60(52–68)
**Gumbo**	27,257	4,167	654	38,103	72	69(63–75)
**All 3 target areas**	140,249	53,543	262	184,068	76	69(64–74)

^1^ Number of people who received OCV in each target based on tally sheets from each vaccination team

^2^ The population estimates generated from the most recent census were considered to vastly underestimate the true number of inhabitants so digital satellite images were used to provide alternative estimates allowing a more conservative planning. Estimates were calculated using the number of built structures divided by 2 (mean number of structures per household from survey) and multiplied by 6 (est. number of individuals per household)

^3^ Calculated by dividing the number of vaccines distributed by the estimated population size. Tally sheets were used to record the number of people vaccinated at each site per sex and age group (1–4 years, 5–14 years, 15 years and over)

^4^Individuals were considered vaccinated regardless of whether they had a vaccination card.

^5^Settlement did not officially exist at the time of the 2008 census

**Table 2:** Number of people who received OCV by age category and sex based on tally sheets from each vaccination team; and estimated vaccine coverage.

	Male	Female	Total	Vaccine Coverage^1^
	N (%)	N (%)	N	% (95%CI)
**Neighborhood-targeted campaign**				
1–4 years	11,170 (50.4)	10,975 (49.6)	22,145	79(72–86)
5–15 years	20,931 (49.3)	21,535 (50.7)	42,466	90(86–94)
≥15 years	39,844 (52.7)	35,794 (47.3)	75,638	53(46–59)
Total	71,945 (51.3)	68,304 (48.7)	140,249	69(64–74)
****Case-triggered interventions****				
1–4 years	2,029 (51.0)	2,062 (49.0)	4,091	65(50–80)
5–15 years	3,943 (50.0)	3,943 (50.0)	7,886	68(57–79)
≥15 years	3,660 (53.6)	5,486 (46.4)	9,146	43(33–52)
Total	9,632 (51.9)	11,491 (48.1)	21,123	51(42–60)

^1^From population-based survey. Individuals were considered vaccinated regardless of whether they had a vaccination card.

**Fig 2:**
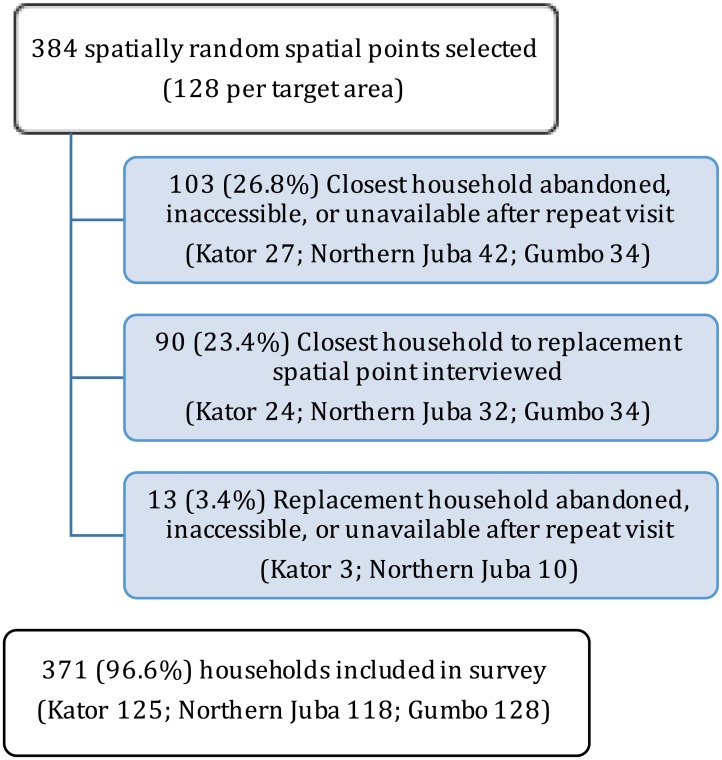
Selection of households for inclusion in the neighborhood-targeted vaccine coverage survey.

**Fig 3:**
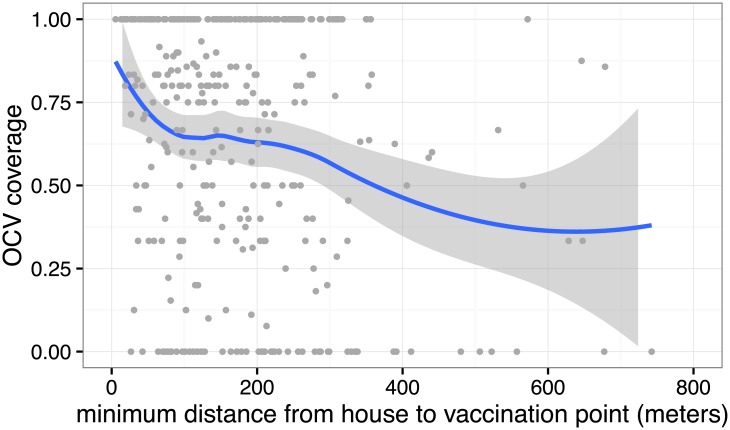
Estimated vaccine coverage by distance to the closest vaccination site in the neighborhood-targeted campaign. Line represents LOESS smoothed estimates of coverage with non-parametric 95% confidence intervals.

A total of 371 households were included in the coverage survey of targeted neighborhoods (Fig 2). No households refused to participate in the survey. Household size varied from 1 to 20 with a median of 6 (S2 Table). The mean number of built structures per household was 2 (S2 Table). All but two households with available coordinates were within 1 kilometer from the closest vaccination site (Fig 3), with a median distance to the closest vaccination site of 156 meters. We ascertained the vaccination status for 96.8% (2578/2662) of individuals, with 94% of those who reported to have been vaccinated providing a vaccination card. We estimated the vaccine coverage (self-report) to range from 60–70% (S1 Table) with an overall population-weighted coverage across the three targeted areas of 68.8% (95% CI: 64.0–73.7, Table 2).

**Fig 4:**
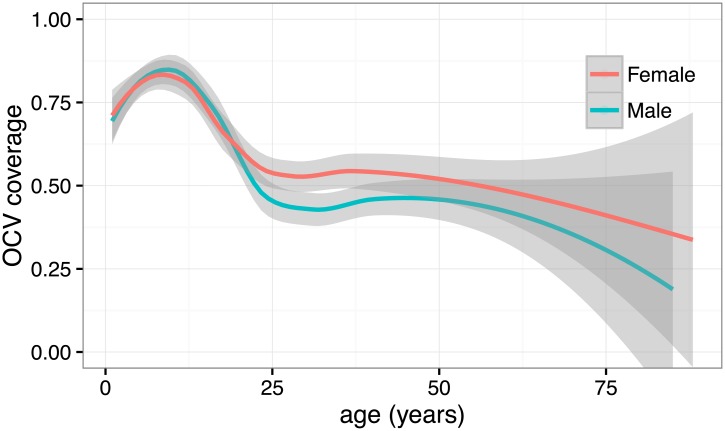
Estimated vaccine coverage by age and sex in the neighborhood-targeted campaign. Red represents women and blue represents men. Lines represent LOESS smoothed estimates of coverage by sex and non-parametric 95% confidence intervals.

Vaccination status between individuals in the same household was more correlated than expected, with a survey design effect of 7.3. In Northern Juba, nearly 1 in 3 households (30%) reported that no household members were vaccinated (S2 Table). The proportion of household members vaccinated decreased with distance to the closest vaccination site (Fig 3). Coverage was highest among children 5–14 years (90.0%; 95% CI: 85.7–94.3). While overall vaccine coverage was similar between women (68.9%; 95% CI: 63.7–74.0) and men (64.7%; 95% CI: 57.7–71.7) on average; adult women tended to have higher coverage than adult men (RR 0.81, 95% CI: 0.68–0.96), with less than half the men 15 years or older reporting to have been vaccinated (Fig 4, S3 Table). The main reasons for non-vaccination in the main campaign were; (1) not being aware of the campaign (256, 30% of unvaccinated individuals), (2) being absent during the time of the campaign (202, 23%), and (3) not having time (129, 15%, S4 Table).

### Case-triggered comprehensive targeted interventions

Of the 54 suspected cholera cases from Juba screened by direct and enriched RDT during the CTI period, 17 were positive by enriched RDT. We carried out CTI at the homes of 14 (82%) of these enriched RDT positive cases. One additional direct RDT positive household (out of 9 direct-RDT positive only) was included in CTI as it occurred on a day with no other priority activities for the teams. The remaining two CTIs occurred around the residence of individuals who had reportedly died due to acute watery diarrhea for whom no sample was available (1 community- and 1 facility-death). Two additional deaths were reported during this period although the team was unaware of these deaths at the time of the activities.

All but two of the CTIs took place in areas that had not been covered in the main campaign. The CTIs occurred 1–6 days after the suspected cholera case had presented at the health facility (mean delay 3.4 days). Ten CTIs were single-day events and the remaining 7 took place over a two-day period. Based on tally sheets collected at the CTI sites, 11,491 (54.4%) of those who received the intervention were female, 4,091(19.4%) were children 1–4 years old and 7,886 (37.3%) were children 4–14 years old.

Coverage surveys were carried out in 13 of 17 CTIs (exact location of the patient’s home was unavailable for 3 and one was outside of Juba town), with a total of 390 individuals sampled. Vaccine coverage per CTI site ranged from 30% (95%CI 12.6–47.4) to 86.7% (95%CI 73.7–99.6). Overall, the coverage was 51.0% (95%CI 41.7–60.3), with no significant difference between those sites targeted inside and outside the main campaign target area. Coverage patterns were like those observed in the main neighborhood-targeted campaign. Adult men were less likely to have received the vaccine compared to adult women (RR 0.73; 95%CI 0.60–0.89). Overall coverage was 45.7% (95%CI 35.5–55.8) among men and 55.8% (95%CI 45.5–66.1) among women. Coverage was also highest among school-aged children (Table 2).

## Discussion

We provided a single-dose of OCV through spatially-targeted campaigns to over 160,000 individuals in Juba, South Sudan. We achieved nearly 70% vaccine coverage within the main, neighborhood-targeted, campaign and had no significant challenges in using a targeted strategy within this large urban setting. Our experience should ease concerns about targeting specific populations with OCV in urban settings, even during an outbreak. Similarly, targeted OCV campaigns have been successfully implemented in urban slums of Haiti [13]. These findings support the possibility of targeting particular neighborhoods that may be responsible for driving urban cholera epidemics, which may provide an efficient way to minimize cost and maximize public health impact [14].

We also demonstrated that it is feasible for multiple actors (e.g., MoH and humanitarian organizations) to work together to rapidly provide a suite of cholera control interventions to the high-risk group living near cholera cases with moderate coverage. While this type of approach is intuitively appropriate for cholera control given the evidence of elevated risk around cases [7,8], evaluations of the effectiveness of similar interventions in the future are needed. Here, the case-triggered CTI approach was used at the end of the outbreak, when cases were sporadic, with the hopes of quelling the outbreak. Consequently, numbers were low and given the approach was hastily devised during the outbreak, there was limited time for detailed planning to optimize impact and to incorporate any detailed evaluation of effectiveness. More work is needed to best define the best mix of components to include in CTI, including the possibility of prophylactic antibiotics, to halt cholera transmission. This approach is not likely to be a silver bullet for cholera control, but may prove to be an efficient strategy in periods of low transmission, perhaps seasonally as has been proposed in Haiti [15], or to accelerate the end of an outbreak after mass campaigns.

Although some OCV campaigns have achieved higher coverage, our estimates are consistent with others in urban areas [16–19]. Despite initial concerns, vaccine sites were not over-run with population from elsewhere in the city, and even in the targeted areas, coverage was less than expected. One potential reason that public interest in vaccine was lower than expected may be due to ‘cholera fatigue,’ where after the much larger 2014 outbreak [3], individuals and the media paid much less attention to cholera in 2015.

While vaccine coverage was lower in adult men, use of the administrative data alone masked this difference and suggested roughly equal OCV coverage by sex. This was especially apparent in Kator where a substantial proportion of those that received the vaccine during the campaign were adult men (53.6% per tally sheets), but little over 40% of the men who lived in the area received the vaccine (S1 and S2 Tables). Being a commercial part of town, it is possible that these were male businessman working in the area during the day but who lived elsewhere in the city.

A door-to-door strategy may have been more appropriate for this highly targeted campaign, however other campaigns using a mixture of fixed sites and door-to-door vaccine delivery report a similar coverage among urban populations and similar challenges reaching adult men [19]. Keeping vaccination sites open later (security situation dependent), and perhaps moving them near places where people congregate in the early evening could prove a useful strategy to improve coverage among men, given that lack of time was the most common reason for non-vaccination. On the other hand, if the reason for not having time was due to work commitments, campaigns could target workplaces during the day. The high coverage among school-aged children likely reflects the success of using schools as vaccination sites, as has been observed in other settings [20].

We observed heavy clustering of vaccination status within households of the main coverage survey. Over 15% of the households sampled had no vaccinated individuals, despite most households visited being well within walking distance from a vaccination site (e.g., more than half being within 160 meters). On the other hand, a third of the households had 100% coverage among eligible members. The most common reason for non-vaccination in the neighborhood campaign was not being aware of the campaign, perhaps reflecting the limited use of radio and other measures to publicize the campaign. Ensuring at least one person in every house is aware of the OCV campaign could be an important approach to increasing overall coverage. A door-to-door strategy for social mobilization rather than for vaccine delivery could help increase household-level knowledge of the campaign.

This experience in Juba highlights the key challenge of designing public health interventions in low-resource, volatile settings such as South Sudan, where accurate and up-to-date demographic information is not always available. The use of satellite imagery has been used to estimate population size in unstable settings [21] and innovative initiatives like MissingMaps (www.missingmaps.org) make the task more feasible, even in the world’s most vulnerable populations. Nevertheless, local information regarding the different observable characteristics of residential and non-residential built structures and the number of persons per built structure are needed to obtain accurate estimates. Developing standardized methods for gathering and sharing information to aid population estimation in low-resource, data-poor settings is a key priority for efficient public health programming.

Our findings come with several limitations. We based our spatial sampling on building density from recent satellite images rather than true population density. It is possible that some areas of the city, especially the most vulnerable, overcrowded areas may have more people but less built structures and could therefore be underrepresented. Furthermore, we interviewed the senior household member for information regarding vaccination status of the other household members in the main coverage survey. This may have led to information bias and contribute to our findings of high intra-household clustering. This also may have led to less precise and accurate estimates of the reasons for non-vaccination within the household.

In conclusion, we showed that targeting OCV in response to an outbreak within a large urban population both to neighborhoods and neighbors of cholera cases is feasible and well accepted by the population. Developing and testing new ways to reach traditionally hard-to-reach groups, including adult men, remains a priority. While cholera continues to strike in complex settings with mobile populations and dynamic security constraints, flexible targeted approaches and alternative dosing schedules, like the one described here, are needed to maximize the potential impact of the vaccine.

## Supporting information

S1 Table Number of people who received OCV in each target area during the neighborhood-targeted campaign in Juba, by age category and sex based on tally sheets from each vaccination team. (DOCX)

S2 Table Characteristics of the vaccine coverage study sample populations. (DOCX)

S3 Table Estimated vaccine coverage per age category, sex and vaccination target area in the neighborhood-targeted campaign. (DOCX)

S4 Table Reasons for non-vaccination provided by individuals who had not received OCV during the main campaign in Juba. (DOCX)

S1 Text Details of coverage survey approach. (DOCX)
