# Supplementary material for: Neighborhood-targeted and case-triggered use of a single dose of oral cholera vaccine in an urban setting: Feasibility and vaccine coverage
Source: PLoS Negl Trop Dis. 2017 Jun 8;11(6):e0005652. doi: 10.1371/journal.pntd.0005652 (PMC5478158; doi:10.1371/journal.pntd.0005652)
Supplement: S1 Table — (DOCX) [file pntd.0005652.s001.docx]

|  | Received OCV during main campaign | | | | |
| --- | --- | --- | --- | --- | --- |
|  | Male, N (%) | | Female, N (%) | | Total, N |
| **Kator** |  |  |  |  |  |
| 1-4 years | 6,816 | (51.0) | 6,548 | (49.0) | 13,364 |
| 5-15 years | 13,638 | (49.2) | 14,098 | (50.8) | 27,736 |
| $\geq$15 years | 27,272 | (53.6) | 23,581 | (46.4) | 50,853 |
| Total | 47,726 | (51.9) | 44,227 | (48.1) | 91,953 |
| **Northern Juba** |  |  |  |  |  |
| 1-4 years | 2,063 | (49.3) | 2,121 | (50.7) | 4,184 |
| 5-15 years | 2,995 | (49.4) | 3,065 | (50.6) | 6,060 |
| $\geq$15years | 5,498 | (50.9) | 5,297 | (49.1) | 10,795 |
| Total | 10,556 | (50.2) | 10,483 | (49.8) | 21,039 |
| **Gumbo** |  |  |  |  |  |
| 1-4 years | 2,291 | (49.8) | 2,306 | (50.2) | 4,597 |
| 5-15 years | 4,298 | (49.6) | 4,372 | (50.4) | 8,670 |
| $\geq$15 years | 7,074 | (50.6) | 6,916 | (49.4) | 13,990 |
| Total | 13,663 | (50.1) | 13,594 | (49.9) | 27,257 |
| **All 3 target areas** |  |  |  |  |  |
| 1-4 years | 11,170 | (50.4) | 10,975 | (49.6) | 22,145 |
| 5-15 years | 20,931 | (49.3) | 21,535 | (50.7) | 42,466 |
| $\geq$15 years | 39,844 | (52.7) | 35,794 | (47.3) | 75,638 |
| Total | 71,945 | (51.3) | 68,304 | (48.7) | 140,249 |
