# Supplementary material for: Neighborhood-targeted and case-triggered use of a single dose of oral cholera vaccine in an urban setting: Feasibility and vaccine coverage
Source: PLoS Negl Trop Dis. 2017 Jun 8;11(6):e0005652. doi: 10.1371/journal.pntd.0005652 (PMC5478158; doi:10.1371/journal.pntd.0005652)
Supplement: S2 Table — (DOCX) [file pntd.0005652.s002.docx]

|  | **Neighborhood-targeted campaign** | | | | **Case-triggered comprehensive targeted interventions** |
| --- | --- | --- | --- | --- | --- |
|  | **Kator** | **Northern Juba** | **Gumbo** | **All 3 target areas** |  |
| **Households - N** | **125** | **118** | **128** | **371** | **390** |
| Household members - N (IQR) | 7 (5–10) | 6 (4-9) | 6.5 (5-9.5) | 6 (5-9) | Not collected |
| Households with available GPS location – N (%) | 121(97%) | 101(86%) | 123(96%) | 345 (93%) | 390(100%) |
| Distance from closest vaccination site, Median (IQR) | 123 (71-198) | 209 (130-288) | 140 (90–224) | 156 (92–242) | Not applicable |
| Households where no one was vaccinated | 16 (13%) | 35 (30%) | 14 (11.4%) | 65 (17%) | Not collected |
| Households with all eligible members vaccinated | 43 (34%) | 39 (33%) | 39 (30%) | 121 (33%) | Not collected |
| Number of huts/dwelling, Median (IQR) | 3 huts (2 – 4) | 2 huts (2-3) | 2 huts (1-3) | 2 huts (2 – 4) | Not collected |
| **Individuals - N** | **906** | **796** | **960** | **2662** | **390** |
| Female (N, %) | 334 (46) | 400 (50) | 505 (53) | 1239 (50) | 206 (53) |
| Age – Median (IQR) | 19 (8-30) | 15 (5-29) | 20 (7-31) | 18 (7-30) | 22 (9-30) |
| 1-4 years – N (%) | 104 (14) | 172 (22) | 151 (16) | 427 (17) | 57 (15) |
| 5-14 years – N (%) | 194 (27) | 215 (27) | 241 (25) | 650 (26) | 78 (20) |
| 15+ years – N (%) | 434 (59) | 409 (51) | 568 (59) | 1411(57) | 255 (65) |
