# Supplementary material for: Neighborhood-targeted and case-triggered use of a single dose of oral cholera vaccine in an urban setting: Feasibility and vaccine coverage
Source: PLoS Negl Trop Dis. 2017 Jun 8;11(6):e0005652. doi: 10.1371/journal.pntd.0005652 (PMC5478158; doi:10.1371/journal.pntd.0005652)
Supplement: S3 Table — (DOCX) [file pntd.0005652.s003.docx]

|  | **Estimated Vaccine Coverage, % (95% CI)** | | | | | | | | | | | |
| --- | --- | --- | --- | --- | --- | --- | --- | --- | --- | --- | --- | --- |
|  | **1 - 4 years** | | | **5 - 15 years** | | | **15 + years** | | | **Total** | | |
|  | **Male** | **Female** | **Total** | **Male** | **Female** | **Total** | **Male** | **Female** | **Total** | **Male** | **Female** | **Total** |
| **Gumbo** | 78(67-89) | 86(76-97) | 82(74-90) | 90(83-96) | 87(81-95) | 89(84-94) | 52(41-63) | 59(51-68) | 56(48-65) | 67(59-74) | 71(64-78) | 69(63-75) |
| **Kator** | 80(67-93) | 84(71-98) | 82(71-93) | 92(87-99) | 95(87-100) | 94(88-100) | 43(32-56) | 58(49-67) | 51(42-60) | 64(54-74) | 70(62-78) | 70(63-77) |
| **Juba North** | 63(49-78) | 60(47-74) | 62(50-75) | 73(61-87) | 66(52-80) | 70(58-82) | 55(44-66) | 52(43-62) | 54(45-62) | 62(53-71) | 58(48-67) | 60(51-68) |
| **All 3 target areas** | 77(68-85) | 81(73-90) | 79(72-86) | 90(85-95) | 89(84-95) | 90(86-94) | 46(38-55) | 58(52-64) | 53(46-59) | 65(58-72) | 69(64-74) | 69(64-74) |
