# Supplementary material for: Neighborhood-targeted and case-triggered use of a single dose of oral cholera vaccine in an urban setting: Feasibility and vaccine coverage
Source: PLoS Negl Trop Dis. 2017 Jun 8;11(6):e0005652. doi: 10.1371/journal.pntd.0005652 (PMC5478158; doi:10.1371/journal.pntd.0005652)
Supplement: S4 Table — (DOCX) [file pntd.0005652.s004.docx]

| **Reason for non-vaccination** | **Kator, N (%)** | **Northern Juba, N (%)** | **Gumbo, N (%)** | **Total, N (%)** |
| --- | --- | --- | --- | --- |
| Not aware of the campaign | 57 (21%) | 157 (50%) | 42 (15%) | 256 (30%) |
| Absent during the campaign | 55 (21%) | 58 (19%) | 89 (32%) | 202 (23%) |
| Did not have time | 51 (21%) | 31 (10%) | 47 (17%) | 129 (15%) |
| Did not know the location of the closest vaccination site | 22 (8%) | 17 (5%) | 21 (7%) | 60 (7%) |
| Was ill during the campaign | 14 (5%) | 6 (2%) | 21 (7%) | 41 (5%) |
| Wait was considered too long | 13 (5%) | 2 (0.6%) | 10 (4%) | 25 (3%) |
| Vaccination site considered to be too far | 5 (2%) | 9 (3%) | 10 (4%) | 24 (3%) |
| Other | 34 (13%) | 35 (11%) | 33 (12%) | 102 (12%) |
| No reason given | 16 (6%) | 2 (0.6%) | 9 (3%) | 27 (3%) |
| **Total unvaccinated** | **267** | **317** | **282** | **861** |
