# Supplementary material for: Neighborhood-targeted and case-triggered use of a single dose of oral cholera vaccine in an urban setting: Feasibility and vaccine coverage
Source: PLoS Negl Trop Dis. 2017 Jun 8;11(6):e0005652. doi: 10.1371/journal.pntd.0005652 (PMC5478158; doi:10.1371/journal.pntd.0005652)
Supplement: S1 Text — (DOCX) [file pntd.0005652.s005.docx]

To assess vaccine coverage in the neighborhood-targeted (main) campaign, a random sample of the population living in each of the target areas was selected using a stratified spatial sampling approach. Households (individuals sleeping under the same roof who share most meals for at least the past two weeks) were the primary sampling units, and sample size was calculated to estimate coverage in each target area with a precision of ±5% using a conservative assumption of 50% coverage, an alpha error of 5%, a design effect of 2 and assuming an average of 6 individuals per household [10]. A total of 128 households were required in each target area.

A proxy for population density was created by enumerating digitized built structures from recent aerial images ([www.openstreetmap.org](http://www.openstreetmap.org)) and aggregating to 250m by 250m grid cells. Three hundred and eighty-four (128 per strata/target area) grid cells were randomly selected with probability proportional to estimated population density, and then randomly selected a point within each selected grid cell. The household nearest to the selected GPS point was included in the survey. All residents 1-year and older at the start of the campaign were eligible for inclusion and vaccination status was ascertained through interviews with the most senior household member present at the time of the household visit. Visual inspection of vaccination cards was used for confirmation, though we classified vaccination status based on self-report. Interviews were conducted in Juba Arabic, or another local language if spoken by both the interviewer and the interviewee. After two failed attempts to contact the members of the selected household, a replacement GPS point was randomly generated to select another household within the same grid cell.

We used a similar approach to estimate coverage in each case-centered targeted intervention cluster and selected 30 spatially random points within 350 meters of suspected case households. As with the population-based survey for the main campaign, the closest household to each GPS point was included in the survey, but instead of ascertaining the vaccination status of all individuals living in the household, one person was selected at random from those residing in (but not necessarily present at the time of the first visit) the household.
